# Supplementary material for: Investigating the relationship between changes in social security benefits and mental health: a protocol for a systematic review
Source: BMJ Open. 2020 Jun 28;10(6):e035993. doi: 10.1136/bmjopen-2019-035993 (PMC7322275; doi:10.1136/bmjopen-2019-035993)
Supplement: Supplementary data [file bmjopen-2019-035993supp002.pdf]

Supplementary File 2

Preliminary search strategy

Medline

1 austerity.mp.

2 welfare benefit.mp.

3 welfare reform.mp.

4 Social assistance.mp.

5 exp Social Security/

6 Social security.mp.

7 Income benefits.mp.

8 Income support.mp.

9 Income supplement.mp.

10 Income maintenance.mp.

11 Pensions/

12 Conditional cash.mp.

13 Cash assistance.mp.

14 Unemployment benefit.mp.

15 Child Benefit.mp.

16 Tax credit.mp.

17 Family benefit.mp.

18 Family support.mp.

19 Conditionality.mp.

20 Poverty reduction.mp.

21 Housing benefit.mp.

22 Anti-poverty.mp.

23 Family allowance.mp.

24 Entitlement.mp.

25 Generosity.mp.

26 Disability benefit.mp.

27 1 or 2 or 3 or 4 or 5 or 6 or 7 or 8 or 9 or 10 or 11 or 12 or 13 or 14 or 15 or 16 or 17 or 18 or 19 or 20 or 21 or 22 or 23 or 24 or 25 or 26

28	Mental.mp.
29	Psychiat*.mp.
30	Distress*.mp.
31	Stress*.mp.
32	Depress*.mp.
33	exp Depression/
34	Wellbeing.mp.
35	Well-being.mp.
36	Anxiety.mp.
37	Quality of life.mp.
38	Mood disorder.mp.
39	Psychological.mp.
40	Suici*.mp.
41	Self-harm.mp.
42	28 or 29 or 30 or 31 or 32 or 33 or 34 or 35 or 36 or 37 or 38 or 39 or 40 or 41
43	Quantitative.mp.
44	Natural experiment.mp.
45	Quasi-experimental.mp.
46	Difference-in-difference.mp.
47	Multi-level.mp.
48	Regression.mp.
49	Longitudinal.mp.
50	Time-series.mp.
51	Logistic.mp.
52	Linear.mp.
53	Poisson.mp.
54	Synthetic control.mp.
55	propensity score.mp.
56	Instrumental variable.mp.
57	Decomposition.mp.

58 Cross-sectional.mp.

59 (before adj2 after).mp.

60 (pre adj2 post).mp.

61 Quasi-natural.mp.

62 Natural Policy Experiment.mp.

63 Confounding.mp.

64 Fixed-effects.mp.

65 43 or 44 or 45 or 46 or 47 or 48 or 49 or 50 or 51 or 52 or 53 or 54 or 55 or 56 or 57 or 58 or 59 or 60 or 61 or 62 or 63 or 64

66 27 and 42 and 65
