# Supplementary material for: Investigating the relationship between changes in social security benefits and mental health: a protocol for a systematic review
Source: BMJ Open. 2020 Jun 28;10(6):e035993. doi: 10.1136/bmjopen-2019-035993 (PMC7322275; doi:10.1136/bmjopen-2019-035993)
Supplement: Author's manuscript [file bmjopen-2019-035993.draft_revisions.pdf]

BMJ Open is committed to open peer review. As part of this commitment we make the peer review history of every article we publish publicly available.

When an article is published we post the peer reviewers' comments and the authors' responses online. We also post the versions of the paper that were used during peer review. These are the versions that the peer review comments apply to.

The versions of the paper that follow are the versions that were submitted during the peer review process. They are not the versions of record or the final published versions. They should not be cited or distributed as the published version of this manuscript.

BMJ Open is an open access journal and the full, final, typeset and author-corrected version of record of the manuscript is available on our site with no access controls, subscription charges or pay-per-view fees (http://bmjopen.bmj.com).

If you have any questions on BMJ Open's open peer review process please email info.bmjopen@bmj.com

BMJ Open

Investigating the relationship between changes in social security benefits and mental health: a protocol for a systematic review

Journal:	*BMJ Open*
Manuscript ID	bmjopen-2019-035993
Article Type:	Protocol
Date Submitted by the Author:	25-Nov-2019

Complete List of Authors:	Simpson, Julija; Newcastle University, Population Health Sciences Institute<br>Brown, Heather ; Newcastle University, Population Health Sciences Institute<br>Bell, Zoe; Newcastle University, Population Health Sciences Institute<br>Bambra, Clare; Newcastle University, Population Health Sciences Institute

Keywords:	MENTAL HEALTH, PUBLIC HEALTH, SOCIAL MEDICINE

SCHOLARONE™ Manuscripts

I, the Submitting Author has the right to grant and does grant on behalf of all authors of the Work (as defined in the below author licence), an exclusive licence and/or a non-exclusive licence for contributions from authors who are: i) UK Crown employees; ii) where BMJ has agreed a CC-BY licence shall apply, and/or iii) in accordance with the terms applicable for US Federal Government officers or employees acting as part of their official duties; on a worldwide, perpetual, irrevocable, royalty-free basis to BMJ Publishing Group Ltd ("BMJ") its licensees and where the relevant Journal is co-owned by BMJ to the co-owners of the Journal, to publish the Work in this journal and any other BMJ products and to exploit all rights, as set out in our licence.

The Submitting Author accepts and understands that any supply made under these terms is made by BMJ to the Submitting Author unless you are acting as an employee on behalf of your employer or a postgraduate student of an affiliated institution which is paying any applicable article publishing charge ("APC") for Open Access articles. Where the Submitting Author wishes to make the Work available on an Open Access basis (and intends to pay the relevant APC), the terms of reuse of such Open Access shall be governed by a Creative Commons licence – details of these licences and which Creative Commons licence will apply to this Work are set out in our licence referred to above.

Other than as permitted in any relevant BMJ Author's Self Archiving Policies, I confirm this Work has not been accepted for publication elsewhere, is not being considered for publication elsewhere and does not duplicate material already published. I confirm all authors consent to publication of this Work and authorise the granting of this licence.

For peer review only

Investigating the relationship between changes in social security benefits and mental health: a protocol for a systematic review

Julija Simpson*[1], Heather Brown[1], Zoe Bell[1] and Clare Bambra[1]

*Corresponding author: Julija Simpson: julija.simpson@newcastle.ac.uk

1 – Population Health Sciences Institute, Newcastle University, Newcastle upon Tyne, NE1 4LP

Word Count: 2,858 (excluding references)

Protocol amendments

In the event of protocol amendments, the date of each amendment will be accompanied by a description of the change and the rationale

For peer review only - http://bmjopen.bmj.com/site/about/guidelines.xhtml

Strengths and limitations of this study

- We employ a rigorous international gold standard methodology of reporting (PRISMA-P) to facilitate the development and reporting of this protocol
- By employing a PRISMA-P guideline this study follows a clear framework to improve the transparency, accuracy and completeness of the systematic review protocol
- To ensure comprehensiveness of the evidence base in the review, we have developed a broad search strategy, supplemented with reference and citation searches as well as hand-searching of key journals
- A potential limitation of this study is that studies may be too heterogeneous to combine effect estimates
- Another limitation is that grey literature will not be included in the review

Abstract

Introduction. Poor mental health is one of the greatest causes of disability in the world. Evidence increasingly shows that population mental health may be influenced by national social security policies. This systematic review aims to establish the relationship between changes to social security policies and mental health in order to help inform recommendations for policy makers, practitioners and future research.

Methods and analysis. A systematic review of quantitative observational studies comparing mental health outcomes before and after changes in social security policies will be conducted. Six major databases, including Medline, PsychInfo, Embase, CINAHL, Proquest and Scopus, will be searched from January 1979 to November 2019. The electronic database searches will be supplemented by reference and citation searches as well as hand-searching of key journals. The outcomes of interest include any objective or subjective mental health outcomes, including stress, anxiety, depression, self-reported mental health scores, subjective wellbeing and suicide. Study selection will follow the Preferred Reporting Items for Systematic Reviews and Meta-Analyses guidelines, and the quality appraisal of the studies will be assessed by the Effective Public Health Project tool. A narrative synthesis will be conducted for all included studies. If data permit, study findings will be synthesised by conducting a meta-analysis.

Ethics and dissemination. As it will be a systematic review, without primary data collection, there will be no requirement for ethical approval. Findings will be disseminated through peer-reviewed publication and in various media, for example, conferences or symposia.

Trial registration number International Prospective Register for Systematic Reviews (PROSPERO) number CRD42019154733.

Keywords

Mental health, social security, welfare reform, systematic review

For peer review only - http://bmjopen.bmj.com/site/about/guidelines.xhtml

Introduction

Mental illness is a major cause of mortality and morbidity worldwide (1). In OECD (Organisation for Economic Cooperation and Development) countries, mental ill-health affects an estimated 20 per cent of the working-age population at any given time, causing major losses to individuals, communities and the economy (2). It has been estimated that the global economy loses about $1 trillion every year in productivity as a result of depression and anxiety (3).

Evidence increasingly shows that mental ill-health is socially determined. (4, 5). The social determinants include "the conditions in which people are born, live, work and age, and the health systems they can access, which are, in turn, shaped by a wider set of forces: economics, social, environmental policies, and politics" (6, p.392).

One of the key policies affecting household income and thus living conditions – particularly those in the lowest socio-economic groups - is social security policy. Broadly defined, social security policy includes income transfers from the state to individuals, with the aim to cover income loss due to poor health and disability, unemployment, low income, single parenthood and old age (7).

A growing body of literature indicates that, while not explicitly designed to, social security policies can have an impact on population mental health. For example, suicide rates in men in the USA fall sharply when individuals become eligible for social security retirement benefits, even though only a small fraction of men retire at this age (8), indicating how providing a safety net can have a strong protective effect on mental health. Lack of social protection, on the other hand, has been associated with negative effects on mental health. In the UK, the austerity-driven changes to the social security system have been associated with increased prevalence of depression (9), suicide (10) and mental health disorders (11).

Although there is a growing body of literature documenting strong links between social disadvantage and mental health problems (12, 13), the evidence base on the health impact of social security reform remains small. A recent umbrella review of social protection policies has identified only two low-quality systematic reviews related to income maintenance policies (14). Both of these reviews focused on specific policies (i.e. unemployment benefits and tax credits) and, to date, the broader range of social security policies and their impacts on mental health has not been reviewed. In the context of global mental health crisis as well as rapidly rising concerns about the impact of recent austerity measures on population health and wellbeing, a better understanding of the relationship between social security reform and mental health is crucial.

The aim of this systematic review is to evaluate the impact of social security reform on mental health and mental health inequalities in high-income countries. By providing robust and concrete evidence, this review will add to the growing evidence base linking social determinants and mental health and will help inform recommendations for policy makers, researchers and areas for further research.

For peer review only - http://bmjopen.bmj.com/site/about/guidelines.xhtml

For peer review only

Methods

The review protocol is reported in accordance with the standards of the Preferred Reporting Items for Systematic Reviews and Meta-analysis Protocols (15) (PRISMA-P, see supplementary file 1).

Preliminary searchers were carried out in October 2019 and the study registered with the PROSPERO International Prospective Register of Systematic Reviews (16) (registration number CRD42019154733) on 31 October 2019. The review is planned to commence November 2019 and is anticipated to take nine months to complete.

Systematic review questions

1. What is the impact of social security reform on mental health?
2. What is the impact of social security reform on mental health and inequalities?

Objectives

This study has two objectives:

1. To systematically review the quantitative evidence of changes in social security benefits on mental health and inequalities.
2. To establish the associational pathways linking social security benefits and mental health.

Eligibility criteria

Studies will be selected according to the criteria outlined in table 1.

Table 1 Eligibility Criteria

	**Inclusion**	**Exclusion**
Participants (P)	All individuals of any age	
Intervention (I)	A national or regional level policy relating to changes in social security benefits	Interventions (i.e. not government policies)<br>Multi-sectorial policies (e.g. Active Labour Market Programmes, broad austerity policies)
Comparison (C)	Before and after (with or without control groups)	
Outcomes (O)	Clearly defined mental health outcomes (e.g. depression, anxiety, stress, suicide), and could include subjective measures as well as symptoms, events and diagnoses.	Non-mental health outcomes (e.g. general health if there is no separate mental health component)
Study type	Non-randomised or uncontrolled trials<br>Prospective and retrospective cohort studies (with and/or without control groups)	Descriptive studies reporting mental health outcomes in benefit recipients vs non recipients (not before and after)<br>Qualitative studies

For peer review only - http://bmjopen.bmj.com/site/about/guidelines.xhtml

	Prospective repeat cross-sectional studies (with and/or with control groups)<br>Ecological studies	Editorial<br>Commentary<br>Expert opinion
Study period	Published in the last 40 years (1979-2019)	Literature published before 1979
Setting	High-income countries (as per World Bank definition*)	Non-high income countries
Study reporting language	English	

*The World Bank classifies as high-income countries those countries with GNI per capita income of $12,375 or more for the current 2019 fiscal year. Further details can be found at http://data.worldbank.org/income-level/OEC.

For peer review only

Interventions

The review will examine a range of social security policies that cover income loss due to poor health and disability, unemployment, low income, single parenthood and old age as well as tax credits and benefits for families with dependent children.

Outcomes

Primary

Studies will be included if one of the outcome measures reported is the number or rate of mental health problems reported. Mental health can be measured both objectively and subjectively. Objective measures include formal diagnoses (e.g. anxiety, depression, suicide). Subjective measures include those that are self-reported (e.g. self-assessed mental health and wellbeing scores, days of good/poor mental health or by validated questionnaires such as Warwick-Edinburgh Mental Health Scale. Due to possible variation in disease definitions over time, we will extract definitions of outcomes as reported in individual studies. We will extract outcomes in all data forms (e.g. dichotomous, continuous) as reported in the included studies.

Studies including general self-assessed health will not be included unless they also report a mental health component. Outcomes reported for specific population subgroups (e.g. by socio-economic status) will also be included. There will be no restrictions on timing of outcomes.

Secondary

Secondary outcomes of this study include mental health inequalities, as measured by socio-economic status.

Study exclusion

Studies will be excluded they were published prior to 1979. The year 1979 was selected as a cut-off date because it marks a significant shift in policy from this period onwards going from passive to active welfare in high-income countries. Only studies in high-income countries will be included.

Study design

Observational study designs will be included if they report mental health outcomes of a national (or regional) social security policy change (before and after) and are based on a quantitative method. This includes ecological designs.

For peer review only - http://bmjopen.bmj.com/site/about/guidelines.xhtml

Search Strategy

We will examine six major databases, including MEDLINE, EMBASE, CINAHL, Proquest, Applied Social Science Index and Abstracts (ASSIA) and PsycINFO from January 1979 until October 2019.

Search terms were identified from scoping searches and comprise three main concepts related to mental health, social security policy change and a quantitative research method. While not exhaustive, the search strategy aims to cover a broad range of social security policies, mental health outcomes and quantitative research methods. An information specialist, trained in systematic reviews, as well as a medical librarian were consulted about designing and piloting the search strategy. A sample search strategy adapted for Medline can be found in Supplementary File 2.

In terms of restrictions, only peer-reviewed English language studies will be included. Electronic database searches will be supplemented by evaluating the references of literature included in the review as well as the references of other major reviews in the field. Citation searches of included articles will be undertaken using the Science and Social Science Citation Indices. Hand-searching of key journals on health and social policy will also be conducted. Grey literature, such as local project reports, will be excluded due to the inherent difficulties in ensuring comprehensive coverage of such texts, and the absence of quality standards.

Study Selection

Studies will be imported to EndNote (version X9) (17) for deduplication and then to an online software programme, Rayyan (18) for screening. One reviewer (JS) will screen all the titles and abstracts, and, given the large numbers of the initial search hits, the second reviewer (ZB) will screen a random sample of 10% of these, with a third reviewer assisting to resolve any disagreements. We will obtain full reports for all titles that appear to meet the inclusion criteria or where there is any uncertainty. If necessary, additional information will be sought by contacting the study authors. Both reviewers will assess the eligibility of the full texts and appraise the quality of the included studies.

Prior to data extraction, a data extraction form will be created and pilot-tested using a sub-set of included studies. The form will be modified based on feedback from data extractors in order to improve the usability and appropriateness of the form. The data abstraction framework will be used as a template for recording significant study characteristics of the literature appropriate for inclusion in the review. This information will include details on: Study author, Year, Title, Population, Mental health outcome measure(s), Policy, Study design, Data source, Data years, Analytic approach and Results. Data will be extracted by two reviewers independently (JS and ZB).

Risk of bias assessment

Quality appraisal will be undertaken by two researchers working independently (JS and ZB), using the Effective Public Health Project (EPHP) tool (19). It includes six component rating sections (selection bias; study design; confounders; blinding; data collection methods; withdrawals and dropouts). Based on the performance in terms of the above criteria, each study will then be assigned a global rating of either 'strong' or 'moderate' or 'weak'. The EPHP tool demonstrates good inter-rater reliability, is applicable across a range of quantitative study types, and is recommended by the Cochrane Public Health Group. Any disagreements between the researchers will be resolved through a discussion between

For peer review only - http://bmjopen.bmj.com/site/about/guidelines.xhtml

reviewers, if necessary with a third reviewer (HB). The quality of the studies will form a part of the narrative synthesis to highlight the variation between studies, and to help demonstrate the overall strength of evidence in this study area.

Data synthesis

Since combining results from individual studies has the advantage of increasing both statistical power and precision in estimating the impact of social security policies, it is beneficial to undertake a meta-analysis of collated studies in circumstances where clinical and methodological homogeneity permit. In the event that sufficient studies of similar construct and outcomes are identified, a meta-analysis of the results will be undertaken. The continuous outcomes relating to mental health will likely be studied by investigation of the policy effect size, through measurement of the standardised mean difference in mental health before and after policy in question. This will be presented in a forest plot, facilitating both visual and statistical comparison. A sensitivity analysis will be undertaken, addressing whether the exclusion of studies with poor quality rating has an impact on the precision and overall conclusions of the review. Heterogeneity will be quantified by measuring the I^2 statistic, and if appropriate, subgroup analysis will be undertaken.

If meta-analysis is not possible, data from collated studies will be presented in a tabulated summary recording the range of policies, outcomes, and populations that have been included in the review, with appropriate grouping according to study characteristics. A clear accompanying descriptive account addressing the robustness of the evidence presented, and the relationships within and between studies included in the review will be provided. Since by definition, narrative synthesis is an inherently more subjective process, it is crucial to utilise a rigorous and transparent approach to the data collected. For this reason, we will use The Economic and Social Research Council framework and guidelines to present the narrative synthesis, as described by Popay and colleagues (20).

Patient and public involvement

We will not involve patients and the public in this review.

Discussion

Strengths and limitations

To our knowledge, this will be the first systematic review to explore the relationship between social security reform and mental health. The wide range of policies included will provide a comprehensive overview of how social security policies can affect mental health. The review will thus provide valuable information for policymakers and will provide insights for areas for future research. By following a systematic and transparent approach, and adhering to recommended and validated methods guidelines, we aim to ensure that our findings present a valid representation of the existing evidence.

There are several potential limitations associated with this review. First, including a wide range of policies and mental health outcomes, may result in a high heterogeneity between included studies which may limit the statistical power to conduct a meta-analysis. Second, our decision to exclude grey literature as well as non-English studies, might exclude some relevant policy evaluations, particularly those outside of Europe, Australia or North America. However, due to resource limitations, this is beyond the scope of our review. Further, as with

For peer review only - http://bmjopen.bmj.com/site/about/guidelines.xhtml

most systematic reviews, our review is susceptible to publication bias whereby only studies with non-null findings are more likely to be published. We aim to minimise this limitation by reviewing the references and citations of the included studies as well as by hand-searching the key journals, in this way ensuring that the broadest range of relevant literature has been identified. The findings of our review may also limited by the nature of study designs of the included studies, as they will be observational. We will however conduct a rigorous quality assessment of each study and will account for study quality in our narrative synthesis.

Ethics and Dissemination

Since this is a review of secondary data, ethical approval will not be needed. The findings from this review will be disseminated by submission to a peer-reviewed journal. The results will also be presented at a national conference and circulated to the general public and key stakeholder groups using social media.

Author Contributions

JS drafted the manuscript and is the guarantor for the review. All authors contributed to the development of the selection criteria, the risk of bias assessment strategy and data extraction criteria. JS developed the search strategy, HB, ZB and CB provided comments and amendments. All authors read, provided feedback and approved the final manuscript.

Funding Statement

This systematic review is funded by the Economic and Social Research Council, as a part of a PhD project. The funder had no role in developing the systematic review protocol.

Conflicts of interest

None declared.

Abbreviations

PRISMA-P: Preferred Reporting Items for Systematic Reviews and Meta-Analyses (for Protocols)

PROSPERO: international prospective register of systematic reviews

Supplementary Files

Supplementary File 1 – completed PRISMA-P checklist

Supplementary File 2 – preliminary search strategy (Medline)

Bibliography

1. Vos T, Barber RM, Bell B, Bertozzi-Villa A, Biryukov S, Bolliger I, et al. Global, regional, and national incidence, prevalence, and years lived with disability for 301 acute and chronic diseases and injuries in 188 countries, 1990–2013: a systematic analysis for the Global Burden of Disease Study 2013. The Lancet. 2015;386(9995):743-800.
2. World Bank Group. Mental Health 2018 [Accessed 20/11/2019]. Available from: https://www.worldbank.org/en/topic/mental-health.
3. Chisholm D, Sweeny K, Sheehan P, Rasmussen B, Smit F, Cuijpers P, et al. Scaling-up treatment of depression and anxiety: a global return on investment analysis. The Lancet Psychiatry. 2016;3(5):415-24.

For peer review only - http://bmjopen.bmj.com/site/about/guidelines.xhtml

4. Allen J, Balfour R, Bell R, Marmot M. Social determinants of mental health. International Review of Psychiatry. 2014;26(4):392-407.
5. Macintyre A, Ferris D, Gonçalves B, Quinn N. What has economics got to do with it? The impact of socioeconomic factors on mental health and the case for collective action. Palgrave Communications. 2018;4(1):10.
6. Marmot M, Friel S, Bell R, Houweling TA, Taylor S, Health CoSDo. Closing the gap in a generation: health equity through action on the social determinants of health. The lancet. 2008;372(9650):1661-9.
7. Bambra C. Work, worklessness, and the political economy of health: Oxford University Press; 2011.
8. DeSimone J. Suicide and the social security early retirement age. Contemporary economic policy. 2018;36(3):435-50.
9. Reeves A, Clair A, McKee M, Stuckler D. Reductions in the United Kingdom's Government Housing Benefit and Symptoms of Depression in Low-Income Households. American Journal of Epidemiology. 2016;184(6):421-9.
10. Barr B, Taylor-Robinson D, Stuckler D, Loopstra R, Reeves A, Whitehead M. 'First, do no harm': are disability assessments associated with adverse trends in mental health? A longitudinal ecological study. Journal of Epidemiology and Community Health. 2016;70(4):339-45.
11. Katikireddi SV, Molaodi OR, Gibson M, Dundas R, Craig P. Effects of restrictions to Income Support on health of lone mothers in the UK: a natural experiment study. Lancet Public Health. 2018;3(7):e333-e40.
12. Fryers T, Melzer D, Jenkins R. Social inequalities and the common mental disorders. Social psychiatry and psychiatric epidemiology. 2003;38(5):229-37.
13. Muntaner C, Eaton WW, Miech R, O'Campo P. Socioeconomic position and major mental disorders. Epidemiologic reviews. 2004;26(1):53-62.
14. Hillier-Brown F, Thomson K, McGowan V, Cairns J, Eikemo TA, Gil-Gonzále D, et al. The effects of social protection policies on health inequalities: evidence from systematic reviews. Scandinavian journal of public health. 2019;47(6):655-65.
15. Shamseer L, Moher D, Clarke M, Ghersi D, Liberati A, Petticrew M, et al. Preferred reporting items for systematic review and meta-analysis protocols (PRISMA-P) 2015: elaboration and explanation. Bmj. 2015;349:g7647.
16. PROSPERO. York, England: Centre for Reviews and Dissemination; University of York; 2019 [Accessed 21/11/2019]. Available from: https://www.crd.york.ac.uk/prospero/.
17. Web of Science Group. Endnote X9 2019 [Accessed 20/11/2019]. Available from: https://endnote.com/].
18. Ouzzani M, Hammady H, Fedorowicz Z, Elmagarmid A. Rayyan—a web and mobile app for systematic reviews. Systematic reviews. 2016;5(1):210.
19. Thomas B, Ciliska D, Dobbins M, Micucci S. A process for systematically reviewing the literature: providing the research evidence for public health nursing interventions. Worldviews on Evidence-Based Nursing. 2004;1(3):176-84.
20. Popay J, Roberts H, Sowden A, Petticrew M, Arai L, Rodgers M, et al. Guidance on the conduct of narrative synthesis in systematic reviews. A product from the ESRC methods programme Version. 2006;1:b92.

For peer review only - http://bmjopen.bmj.com/site/about/guidelines.xhtml

Reporting checklist for protocol of a systematic review.

Based on the PRISMA-P guidelines.

Instructions to authors

Complete this checklist by entering the page numbers from your manuscript where readers will find each of the items listed below.

Your article may not currently address all the items on the checklist. Please modify your text to include the missing information. If you are certain that an item does not apply, please write "n/a" and provide a short explanation.

Upload your completed checklist as an extra file when you submit to a journal.

In your methods section, say that you used the PRISMA-Preporting guidelines, and cite them as:

Moher D, Shamseer L, Clarke M, Ghersi D, Liberati A, Petticrew M, Shekelle P, Stewart LA. Preferred Reporting Items for Systematic Review and Meta-Analysis Protocols (PRISMA-P) 2015 statement. Syst Rev. 2015;4(1):1.

		Reporting Item	Page Number
**Title**			
Identification	#1a	Identify the report as a protocol of a systematic review	1
Update	#1b	If the protocol is for an update of a previous systematic review, identify as such	1

For peer review only

**Registration**			
	#2	If registered, provide the name of the registry (such as PROSPERO) and registration number	1
**Authors**			
Contact	#3a	Provide name, institutional affiliation, e-mail address of all protocol authors; provide physical mailing address of corresponding author	1
Contribution	#3b	Describe contributions of protocol authors and identify the guarantor of the review	8
**Amendments**			
	#4	If the protocol represents an amendment of a previously completed or published protocol, identify as such and list changes; otherwise, state plan for documenting important protocol amendments	1
**Support**			
Sources	#5a	Indicate sources of financial or other support for the review	8
Sponsor	#5b	Provide name for the review funder and / or sponsor	8
Role of sponsor or funder	#5c	Describe roles of funder(s), sponsor(s), and / or institution(s), if any, in developing the protocol	8
**Introduction**			

For peer review only

Rationale	#6	Describe the rationale for the review in the context of what is already known	3
Objectives	#7	Provide an explicit statement of the question(s) the review will address with reference to participants, interventions, comparators, and outcomes (PICO)	4
**Methods**			
Eligibility criteria	#8	Specify the study characteristics (such as PICO, study design, setting, time frame) and report characteristics (such as years considered, language, publication status) to be used as criteria for eligibility for the review	4,5
Information sources	#9	Describe all intended information sources (such as electronic databases, contact with study authors, trial registers or other grey literature sources) with planned dates of coverage	5
Search strategy	#10	Present draft of search strategy to be used for at least one electronic database, including planned limits, such that it could be repeated	9
Study records - data management	#11a	Describe the mechanism(s) that will be used to manage records and data throughout the review	6
Study records - selection process	#11b	State the process that will be used for selecting studies (such as two independent reviewers) through each phase of the review (that is, screening, eligibility and inclusion in meta-analysis)	6

For peer review only

Study records - data collection process	#11c	Describe planned method of extracting data from reports (such as piloting forms, done independently, in duplicate), any processes for obtaining and confirming data from investigators	6
Data items	#12	List and define all variables for which data will be sought (such as PICO items, funding sources), any pre-planned data assumptions and simplifications	7
Outcomes and prioritization	#13	List and define all outcomes for which data will be sought, including prioritization of main and additional outcomes, with rationale	5,6
Risk of bias in individual studies	#14	Describe anticipated methods for assessing risk of bias of individual studies, including whether this will be done at the outcome or study level, or both; state how this information will be used in data synthesis	7
Data synthesis	#15a	Describe criteria under which study data will be quantitatively synthesised	7
Data synthesis	#15b	If data are appropriate for quantitative synthesis, describe planned summary measures, methods of handling data and methods of combining data from studies, including any planned exploration of consistency (such as I2, Kendall's τ)	7
Data synthesis	#15c	Describe any proposed additional analyses (such as sensitivity or subgroup analyses, meta-regression)	7

For peer review only

Data synthesis	#15d	If quantitative synthesis is not appropriate, describe the type of summary planned	7
Meta-bias(es)	#16	Specify any planned assessment of meta-bias(es) (such as publication bias across studies, selective reporting within studies)	8
Confidence in cumulative evidence	#17	Describe how the strength of the body of evidence will be assessed (such as GRADE)	7

The PRISMA-P checklist is distributed under the terms of the Creative Commons Attribution License CC-BY 4.0. This checklist was completed on 25. November 2019 using https://www.goodreports.org/, a tool made by the EQUATOR Network in collaboration with Penelope.ai

For peer review only

Supplementary File 2

Preliminary search strategy

Medline

1 austerity.mp.

2 welfare benefit.mp.

3 welfare reform.mp.

4 Social assistance.mp.

5 exp Social Security/

6 Social security.mp.

7 Income benefits.mp.

8 Income support.mp.

9 Income supplement.mp.

10 Income maintenance.mp.

11 Pensions/

12 Conditional cash.mp.

13 Cash assistance.mp.

14 Unemployment benefit.mp.

15 Child Benefit.mp.

16 Tax credit.mp.

17 Family benefit.mp.

18 Family support.mp.

19 Conditionality.mp.

20 Poverty reduction.mp.

21 Housing benefit.mp.

22 Anti-poverty.mp.

23 Family allowance.mp.

24 Entitlement.mp.

25 Generosity.mp.

26 Disability benefit.mp.

27 1 or 2 or 3 or 4 or 5 or 6 or 7 or 8 or 9 or 10 or 11 or 12 or 13 or 14 or 15 or 16 or 17 or 18 or 19 or 20 or 21 or 22 or 23 or 24 or 25 or 26

For peer review only

28 Mental.mp.

29 Psychiat*.mp.

30 Distress*.mp.

31 Stress*.mp.

32 Depress*.mp.

33 exp Depression/

34 Wellbeing.mp.

35 Well-being.mp.

36 Anxiety.mp.

37 Quality of life.mp.

38 Mood disorder.mp.

39 Psychological.mp.

40 Suici*.mp.

41 Self-harm.mp.

42 28 or 29 or 30 or 31 or 32 or 33 or 34 or 35 or 36 or 37 or 38 or 39 or 40 or 41

43 Quantitative.mp.

44 Natural experiment.mp.

45 Quasi-experimental.mp.

46 Difference-in-difference.mp.

47 Multi-level.mp.

48 Regression.mp.

49 Longitudinal.mp.

50 Time-series.mp.

51 Logistic.mp.

52 Linear.mp.

53 Poisson.mp.

54 Synthetic control.mp.

55 propensity score.mp.

56 Instrumental variable.mp.

57 Decomposition.mp.

For peer review only

58 Cross-sectional.mp.

59 (before adj2 after).mp.

60 (pre adj2 post).mp.

61 Quasi-natural.mp.

62 Natural Policy Experiment.mp.

63 Confounding.mp.

64 Fixed-effects.mp.

65 43 or 44 or 45 or 46 or 47 or 48 or 49 or 50 or 51 or 52 or 53 or 54 or 55 or 56 or 57 or 58 or 59 or 60 or 61 or 62 or 63 or 64

66 27 and 42 and 65

For peer review only

Investigating the relationship between changes in social security benefits and mental health: a protocol for a systematic review

Journal:	*BMJ Open*
Manuscript ID	bmjopen-2019-035993.R1
Article Type:	Protocol
Date Submitted by the Author:	11-Feb-2020
Complete List of Authors:	Simpson, Julija; Newcastle University, Population Health Sciences Institute<br>Brown, Heather ; Newcastle University, Population Health Sciences Institute<br>Bell, Zoe; Newcastle University, Population Health Sciences Institute<br>Bambra, Clare; Newcastle University, Population Health Sciences Institute
**Primary Subject Heading**:	Public health
Secondary Subject Heading:	Mental health
Keywords:	PUBLIC HEALTH, SOCIAL MEDICINE, MENTAL HEALTH

I, the Submitting Author has the right to grant and does grant on behalf of all authors of the Work (as defined in the below author licence), an exclusive licence and/or a non-exclusive licence for contributions from authors who are: i) UK Crown employees; ii) where BMJ has agreed a CC-BY licence shall apply, and/or iii) in accordance with the terms applicable for US Federal Government officers or employees acting as part of their official duties; on a worldwide, perpetual, irrevocable, royalty-free basis to BMJ Publishing Group Ltd ("BMJ") its licensees and where the relevant Journal is co-owned by BMJ to the co-owners of the Journal, to publish the Work in this journal and any other BMJ products and to exploit all rights, as set out in our licence.

The Submitting Author accepts and understands that any supply made under these terms is made by BMJ to the Submitting Author unless you are acting as an employee on behalf of your employer or a postgraduate student of an affiliated institution which is paying any applicable article publishing charge ("APC") for Open Access articles. Where the Submitting Author wishes to make the Work available on an Open Access basis (and intends to pay the relevant APC), the terms of reuse of such Open Access shall be governed by a Creative Commons licence – details of these licences and which Creative Commons licence will apply to this Work are set out in our licence referred to above.

Other than as permitted in any relevant BMJ Author's Self Archiving Policies, I confirm this Work has not been accepted for publication elsewhere, is not being considered for publication elsewhere and does not duplicate material already published. I confirm all authors consent to publication of this Work and authorise the granting of this licence.

For peer review only

Investigating the relationship between changes in social security benefits and mental health: a protocol for a systematic review

Julija Simpson*[1], Heather Brown[1], Zoe Bell[1] and Clare Bambra[1]

*Corresponding author: Julija Simpson: julija.stoniute@newcastle.ac.uk

1 – Population Health Sciences Institute, Newcastle University, Newcastle upon Tyne, NE1 4LP

Word Count: 3,283 (excluding references)

Protocol amendments

In the event of protocol amendments, the date of each amendment will be accompanied by a description of the change and the rationale

For peer review only - http://bmjopen.bmj.com/site/about/guidelines.xhtml

Abstract

Introduction. Poor mental health is one of the greatest causes of disability in the world. Evidence increasingly shows that population mental health may be influenced by national social security policies. This systematic review aims to establish the relationship between social security and mental health in order to help inform recommendations for policy makers, practitioners and future research.

Methods and analysis. A systematic review of quantitative observational studies comparing mental health outcomes before and after changes in social security policies will be conducted. Six major databases, including Medline, PsychInfo, Embase, CINAHL, Proquest and Scopus, will be searched from January 1979 to February 2020. The electronic database searches will be supplemented by reference and citation searches as well as hand-searching of key journals. The outcomes of interest are objective or subjective mental health outcomes, including stress, anxiety, depression, self-reported mental health scores, subjective well-being and suicide. Study selection will follow the Preferred Reporting Items for Systematic Reviews and Meta-Analyses guidelines, and the quality of the studies will be assessed by the Validity Assessment Framework designed for appraising econometric studies. A narrative synthesis will be conducted for all included studies. If data permit, study findings will be synthesised by conducting a meta-analysis.

Ethics and dissemination. As it will be a systematic review, without primary data collection, there will be no requirement for ethical approval. Findings will be disseminated through peer-reviewed publication and in various media, for example, conferences or symposia.

Trial registration number International Prospective Register for Systematic Reviews (PROSPERO) number CRD42019154733.

Strengths and limitations of this study

- We employ a rigorous international gold standard methodology of reporting (PRISMA-P) to facilitate the development and reporting of this protocol
- By employing a PRISMA-P guideline this study follows a clear framework to improve the transparency, accuracy and completeness of the systematic review protocol
- To ensure comprehensiveness of the evidence base in the review, we have developed a broad search strategy, supplemented with reference and citation searches as well as hand-searching of key journals
- A potential limitation of this study is that studies may be too heterogeneous to combine effect estimates

Keywords

Mental health, social security, welfare reform, systematic review

Introduction

Mental illness is a major cause of mortality and morbidity worldwide (1). In OECD (Organisation for Economic Cooperation and Development) countries, mental ill-health

For peer review only - http://bmjopen.bmj.com/site/about/guidelines.xhtml

affects an estimated 20 per cent of the working-age population at any given time, causing major losses to individuals, communities and the economy (2). It has been estimated that the global economy loses about $1 trillion every year in productivity as a result of depression and anxiety (3).

Evidence increasingly shows that mental ill-health is socially determined. (4, 5). The social determinants include "the conditions in which people are born, live, work and age, and the health systems they can access, which are, in turn, shaped by a wider set of forces: economics, social, environmental policies, and politics" (6, p.392).

One of the key policies affecting household income and thus living conditions – particularly those in the lowest socio-economic groups - is social security policy. Broadly defined, social security policy includes income transfers from the state to individuals, with the aim to cover income loss due to poor health and disability, unemployment, low income, single parenthood and old age (7).

A growing body of literature indicates that, while not explicitly designed to, social security policies can have an impact on population mental health. For example, suicide rates in men in the USA fall sharply when individuals become eligible for social security retirement benefits, even though only a small fraction of men retire at this age (8). Moreover, two major systematic reviews investigating the relationship between income and health in OECD countries also found that additional financial resources, including those from social security benefits, can have positive effects on both adult (9) and child mental health (10, 11). Lack of social protection, on the other hand, has been associated with negative effects on mental health. In the UK, the austerity-driven changes to the social security system have been associated with increased prevalence of depression (12), suicide (13) and mental health disorders (14).

Although there is a growing body of literature documenting strong links between social disadvantage and mental health problems (15, 16), the evidence base on the health impact of social security reform remains small. A recent umbrella review of social protection policies in OECD countries has identified only two low-quality systematic reviews related to income maintenance policies (17). Both of these reviews focused on specific policies (i.e. unemployment benefits and tax credits) and, to date, the broader range of social security policies and their impacts on mental health has not been reviewed. Furthermore, even though there have been broad systematic reviews of conditional cash transfers in low- and middle-income countries (18, 19), to our knowledge, none of these reviews included mental health as an outcome. Therefore, in the context of global mental health crisis as well as rapidly rising concerns about the impact of recent austerity measures on population health and wellbeing, there is a growing need to better understand the relationship between social security reform and mental health.

The aim of this systematic review is to evaluate the impact of social security reform on mental health and mental health inequalities in high-income countries. By providing robust and concrete evidence, this review will add to the growing evidence base linking social determinants and mental health and will help inform recommendations for policy makers, researchers and areas for further research.

For peer review only - http://bmjopen.bmj.com/site/about/guidelines.xhtml

Methods

The review protocol is reported in accordance with the standards of the Preferred Reporting Items for Systematic Reviews and Meta-analysis Protocols (20) (PRISMA-P, see supplementary file 1).

Preliminary searchers were carried out in October 2019 and the study registered with the PROSPERO International Prospective Register of Systematic Reviews (21) (registration number CRD42019154733) on 31 October 2019. The review is planned to commence February 2020 and is anticipated to take nine months to complete.

Systematic review questions

1. What is the impact of social security reform on mental health?
2. What is the impact of social security reform on mental health and mental health inequalities?

Mental health inequalities are defined as differences in mental health effects by commonly used indicators of socio-economic status such as individual income, wealth, poverty, education level, occupational status, welfare benefit receipt; as well as area-level economic indicators and ethnicity given the strong relationship between ethnicity and lower SES particularly in the USA (22) – as defined by Hillier-Brown et al (17).

Objectives

This study has two objectives:

1. To systematically review the quantitative evidence of changes in social security benefits on mental health and mental health inequalities;
2. To establish the associational pathways linking social security benefits and mental health.

Eligibility criteria

Studies will be selected according to the criteria outlined in table 1.

Table 1 Eligibility Criteria

	**Inclusion**	**Exclusion**
Participants (P)	All individuals of any age	
Intervention (I)	A national or regional level social security reform or a series of reforms, defined as any government change to:<br>1. Cash benefit levels;<br>2. Eligibility/conditionality related to benefit receipt (including changes in eligibility assessment policies);<br>3. Introduction or elimination of an existing benefit policy.	Interventions (i.e. not government policies such as employer-funded insurance schemes)<br><br>Multi-sectorial policies (e.g. Active Labour Market Programmes, broad austerity policies, welfare regimes)<br><br>Policies not considering cash transfers (e.g. in-kind benefits)

For peer review only - http://bmjopen.bmj.com/site/about/guidelines.xhtml

Comparison (C)	Before and after (with or without control groups).	
Outcomes (O)	Clearly defined mental health outcomes (e.g. depression, anxiety, stress, suicide), and could include subjective measures, such as subjective well-being and life satisfaction, as well as symptoms, events and diagnoses.	Non-mental health outcomes (e.g. general health if there is no separate mental health component)<br><br>Outcomes related to health-care utilisation (e.g. hospital admissions, anti-depressant prescriptions)
Study type	Observational 'before and after' studies, including:<br><br>Quasi-experimental studies<br><br>Prospective and retrospective cohort studies (with and/or without control groups)<br><br>Prospective repeat cross-sectional studies (with and/or with control groups)<br><br>Ecological studies	Descriptive studies reporting mental health outcomes in benefit recipients vs non recipients (not before and after)<br><br>Experimental studies<br><br>Qualitative studies<br><br>Editorials<br><br>Commentaries<br><br>Expert opinion articles
Study period	Published in the last 40 years (1979-2019)	Literature published before 1979
Setting	High-income countries (as per World Bank definition*)	Non-high income countries
Study reporting language	English	

For peer review only

Interventions

The review will examine a range of social security policies that cover income loss due to poor health and disability, unemployment, low income, single parenthood and old age as well as tax credits and benefits for families with dependent children.

Outcomes

Primary

Studies will be included if one of the outcome measures reported is the number or rate of mental health problems reported. Mental health can be measured both objectively and subjectively. Objective measures include formal diagnoses (e.g. anxiety, depression, suicide). Subjective measures include those that are self-reported (e.g. self-assessed mental health and wellbeing scores, days of good/poor mental health or by validated questionnaires such as Warwick-Edinburgh Mental Health Scale. Outcomes will be collected as reported. Due to

For peer review only - http://bmjopen.bmj.com/site/about/guidelines.xhtml

possible variation in disease definitions over time, we will extract definitions of outcomes as reported in individual studies. We will extract outcomes in all data forms (e.g. dichotomous, continuous) as reported in the included studies.

Studies including general self-assessed health will not be included unless they also report a mental health component. Outcomes reported for specific population subgroups (e.g. by socio-economic status) will also be included. There will be no restrictions on timing of outcomes.

Secondary

Secondary outcomes of this study include mental health inequalities, as measured by socio-economic status.

Study exclusion

Studies will be excluded they were published prior to 1979. The year 1979 was selected as a cut-off date because it marks a significant shift in policy from this period onwards going from passive to active welfare in high-income countries. Only studies in high income countries will be included.

Study design

Observational study designs will be included if they report mental health outcomes of a national (or regional) social security policy change (before and after) and are based on a quantitative method. This includes ecological designs.

Search Strategy

We will examine seven major databases, including MEDLINE, EMBASE, CINAHL, Proquest, Applied Social Science Index and Abstracts (ASSIA), PsycINFO and Research Papers in Economics from January 1979 until October 2019.

Search terms were identified from scoping searches and comprise three main concepts related to mental health, social security policy change and a quantitative research method. While not exhaustive, the search strategy aims to cover a broad range of social security policies, mental health outcomes and quantitative research methods. An information specialist, trained in systematic reviews, as well as a medical librarian were consulted about designing and piloting the search strategy. A sample search strategy adapted for Medline can be found in Supplementary File 2.

In terms of restrictions, only peer-reviewed English language studies will be included. One exception includes working-papers in the field of Economics, as they may comprise a large proportion of relevant literature. Electronic database searches will be supplemented by evaluating the references of literature included in the review as well as the references of other major reviews in the field. Citation searches of included articles will be undertaken using the Science and Social Science Citation Indices. Hand-searching of key journals on health and social policy will also be conducted.

Study Selection

Studies will be imported to EndNote (version X9) (23) for deduplication and then to an online software programme, Rayyan (24) for screening. One reviewer (JS) will screen all the titles and abstracts, and, given the large numbers of the initial search hits, the second reviewer (ZB) will screen a random sample of 10% of these, with a third reviewer assisting to resolve

For peer review only - http://bmjopen.bmj.com/site/about/guidelines.xhtml

any disagreements. We will obtain full reports for all titles that appear to meet the inclusion criteria or where there is any uncertainty. If necessary, additional information will be sought by contacting the study authors. Both reviewers will assess the eligibility of the full texts and appraise the quality of the included studies.

Prior to data extraction, a data extraction form will be created and pilot-tested using a sub-set of included studies. The form will be modified based on feedback from data extractors in order to improve the usability and appropriateness of the form. The data abstraction framework will be used as a template for recording significant study characteristics of the literature appropriate for inclusion in the review. This information will include details on: Study author, Year, Title, Population, Mental health outcome measure(s), Policy, Study design, Data source, Data years, Analytic approach and Results. Data will be extracted by two reviewers independently (JS and HB).

Risk of bias assessment

Quality appraisal will be undertaken by two researchers working independently (JS and HB), using the Validity Assessment framework developed by Barr and colleagues (25). It includes nine component rating sections (unit of analysis; comparison approach; sample selection; number of time points of data; response bias; exogeneity of policy exposure, confounding, sample size/power and statistical methods). Based on the performance in terms of the above criteria, each study will be assigned a global score and classed as either 'strong' or 'moderate' or 'weak'. Any disagreements between the researchers will be resolved through a discussion between reviewers, if necessary with a third reviewer (ZB). The quality of the studies will form a part of the narrative synthesis to highlight the variation between studies, and to help demonstrate the overall strength of evidence in this study area.

Data synthesis

Since combining results from individual studies has the advantage of increasing both statistical power and precision in estimating the impact of social security policies, it is beneficial to undertake a meta-analysis of collated studies in circumstances where clinical and methodological homogeneity permit. In the event that sufficient studies of similar construct and outcomes are identified, a meta-analysis of the results will be undertaken. The continuous outcomes relating to mental health will likely be studied by investigation of the policy effect size, through measurement of the standardised mean difference in mental health before and after policy in question. This will be presented in a forest plot, facilitating both visual and statistical comparison. A sensitivity analysis will be undertaken, addressing whether the exclusion of studies with poor quality rating has an impact on the precision and overall conclusions of the review. This will be done by excluding one paper at a time and exploring the impact on the overall results. Heterogeneity will be quantified by measuring the I^2 statistic (I^2 statistic with values of 30 to 60%, 50 to 90% and 75 to 100% used to denote moderate, substantial and considerable levels of heterogeneity, respectively (26)). If appropriate, heterogeneity will be further explored by undertaking sub-group analysis. Planned subgroup analyses include those by age, gender and type of policy reform. Publication bias will be assessed using funnel plots, as well as Egger's regression test (27).

If meta-analysis is not possible, mental health effects of each policy will be summarised narratively as follows. First, policies will be classified into five broad categories of social security benefits (i.e. Social Assistance; Families with Children; Disability; Unemployment;

For peer review only - http://bmjopen.bmj.com/site/about/guidelines.xhtml

and Retirement). Policies will be further sub-divided by type of reform – as changes to either benefit levels, eligibility or introduction/elimination of policy. Different magnitudes of each policy will be taken into account by assigning them differential weights, using a modified version of 'direct weighting' approach recommended the Cochrane group (28). This will entail assigning each policy different weights depending on: affected population size; change in magnitude in terms of either benefit level, eligibility requirements or the scale of the policy introduced/removed; risk of bias in each study. The direction of effects will be broadly summarised as either more restrictive or generous policy.

In addition, we will present a tabulated summary of key study characteristics which will include a description of policies, outcomes, populations and settings. This will be followed by a clear accompanying descriptive account addressing the robustness of the evidence presented, and the relationships within and between studies included in the review. Since by definition, narrative synthesis is an inherently more subjective process, it is crucial to utilise a rigorous and transparent approach to the data collected. For this reason, we will use The Economic and Social Research Council framework and guidelines to present the narrative synthesis, as described by Popay and colleagues (29). More specifically, this will include developing a logic model presenting the potential pathways and mediating factors between changes in social security benefits and mental health (e.g. income and employment). As such, the logic model will help guide the structure and interpretation of the findings of the review.

Patient and public involvement

We will not involve patients and the public in this review.

Discussion

Strengths and limitations

To our knowledge, this will be the first systematic review to explore the relationship between social security reform and mental health. Building on a number of successful reviews linking social determinants and health (17, 30-32), the wide range of policies included will provide a comprehensive overview of how social security policies can affect mental health. In our view, taking a comprehensive overview is important because social security or 'welfare' reform usually constitutes a number of policies and thus cannot be reduced to a single intervention. The review will therefore provide valuable information for policymakers as well as insights for areas for future research. In particular, it will help identify gaps in terms of what types of policies require further investigation. Lastly, by following a systematic and transparent approach, and adhering to recommended and validated methods guidelines, we aim to ensure that our findings present a valid representation of the existing evidence.

There are several potential limitations associated with this review. First, including a wide range of policies and mental health outcomes, may result in a high heterogeneity between included studies which may limit the statistical power to conduct a meta-analysis. Second, our decision to exclude non-English studies, might exclude some relevant policy evaluations, particularly those outside of Europe, Australia or North America. However, due to resource limitations, this is beyond the scope of our review. Further, as with most systematic reviews, our review is susceptible to publication bias whereby only studies with non-null findings are more likely to be published. We aim to minimise this limitation by reviewing the references and citations of the included studies as well as by hand-searching the key journals and

For peer review only - http://bmjopen.bmj.com/site/about/guidelines.xhtml

including economic working papers, in this way ensuring that the broadest range of relevant literature has been identified. The findings of our review may also limited by the nature of study designs of the included studies, as they will be observational. We will however conduct a rigorous quality assessment of each study and will account for study quality in our narrative synthesis.

Ethics and Dissemination

Since this is a review of secondary data, ethical approval will not be needed. The findings from this review will be disseminated by submission to a peer-reviewed journal. The results will also be presented at a national conference and circulated to the general public and key stakeholder groups using social media.

Author Contributions

JS drafted the manuscript and is the guarantor for the review. All authors contributed to the development of the selection criteria, the risk of bias assessment strategy and data extraction criteria. JS developed the search strategy, HB, ZB and CB provided comments and amendments. All authors read, provided feedback and approved the final manuscript.

Funding Statement

This systematic review is funded by the Economic and Social Research Council, as a part of a PhD project. The funder had no role in developing the systematic review protocol.

Conflicts of interest

None declared.

Abbreviations

PRISMA-P: Preferred Reporting Items for Systematic Reviews and Meta-Analyses (for Protocols)

PROSPERO: international prospective register of systematic reviews

Supplementary Files

Supplementary File 1 – completed PRISMA-P checklist

Supplementary File 2 – preliminary search strategy (Medline)

Bibliography

1. Vos T, Barber RM, Bell B, Bertozzi-Villa A, Biryukov S, Bolliger I, et al. Global, regional, and national incidence, prevalence, and years lived with disability for 301 acute and chronic diseases and injuries in 188 countries, 1990–2013: a systematic analysis for the Global Burden of Disease Study 2013. The Lancet. 2015;386(9995):743-800.
2. World Bank Group. Mental Health 2018. Available from: https://www.worldbank.org/en/topic/mental-health (accessed 11/02/2020).
3. Chisholm D, Sweeny K, Sheehan P, Rasmussen B, Smit F, Cuijpers P, et al. Scaling-up treatment of depression and anxiety: a global return on investment analysis. The Lancet Psychiatry. 2016;3(5):415-24.

For peer review only - http://bmjopen.bmj.com/site/about/guidelines.xhtml

4. Allen J, Balfour R, Bell R, Marmot M. Social determinants of mental health. International Review of Psychiatry. 2014;26(4):392-407.
5. Macintyre A, Ferris D, Gonçalves B, Quinn N. What has economics got to do with it? The impact of socioeconomic factors on mental health and the case for collective action. Palgrave Communications. 2018;4(1):10.
6. Marmot M, Friel S, Bell R, Houweling TA, Taylor S, Health Commission on Social Determinants of Health. Closing the gap in a generation: health equity through action on the social determinants of health. The Lancet. 2008;372(9650):1661-9.
7. Bambra C. Work, worklessness, and the political economy of health: Oxford University Press; 2011.
8. DeSimone J. Suicide and the social security early retirement age. Contemporary economic policy. 2018;36(3):435-50.
9. Cooper K, Stewart K. Does money in adulthood affect adult outcomes. 2015.
10. Cooper K, Stewart K. Does money affect children's outcomes? 2013.
11. Cooper K. Kitty Stewart Does Money Affect Children's Outcomes? An update. 2017.
12. Reeves A, Clair A, McKee M, Stuckler D. Reductions in the United Kingdom's Government Housing Benefit and Symptoms of Depression in Low-Income Households. American Journal of Epidemiology. 2016;184(6):421-9.
13. Barr B, Taylor-Robinson D, Stuckler D, Loopstra R, Reeves A, Whitehead M. 'First, do no harm': are disability assessments associated with adverse trends in mental health? A longitudinal ecological study. Journal of Epidemiology and Community Health. 2016;70(4):339-45.
14. Katikireddi SV, Molaodi OR, Gibson M, Dundas R, Craig P. Effects of restrictions to Income Support on health of lone mothers in the UK: a natural experiment study. Lancet Public Health. 2018;3(7):e333-e40.
15. Fryers T, Melzer D, Jenkins R. Social inequalities and the common mental disorders. Social psychiatry and psychiatric epidemiology. 2003;38(5):229-37.
16. Muntaner C, Eaton WW, Miech R, O'Campo P. Socioeconomic position and major mental disorders. Epidemiologic reviews. 2004;26(1):53-62.
17. Hillier-Brown F, Thomson K, McGowan V, Cairns J, Eikemo TA, Gil-Gonzále D, et al. The effects of social protection policies on health inequalities: evidence from systematic reviews. Scandinavian journal of public health. 2019;47(6):655-65.
18. Bastagli F, Hagen-Zanker J, Harman L, Barca V, Sturge G, Schmidt T. The impact of cash transfers: A review of the evidence from low-and middle-income countries. Journal of Social Policy. 2019;48(3):569-94.
19. Lagarde M, Haines A, Palmer N. The impact of conditional cash transfers on health outcomes and use of health services in low and middle income countries. The Cochrane database of systematic reviews. 2009(4):Cd008137.
20. Shamseer L, Moher D, Clarke M, Ghersi D, Liberati A, Petticrew M, et al. Preferred reporting items for systematic review and meta-analysis protocols (PRISMA-P) 2015: elaboration and explanation. Bmj. 2015;349:g7647.
21. PROSPERO. York, England: Centre for Reviews and Dissemination; University of York; 2019. Available from: https://www.crd.york.ac.uk/prospero/ (accessed 11/02/2020).
22. Williams DR, Collins C. US socioeconomic and racial differences in health: patterns and explanations. Annual review of sociology. 1995;21(1):349-86.
23. Web of Science Group. Endnote X9 2019. Available from: https://endnote.com/(accessed 11/02/2020).
24. Ouzzani M, Hammady H, Fedorowicz Z, Elmagarmid A. Rayyan—a web and mobile app for systematic reviews. Systematic reviews. 2016;5(1):210.

For peer review only - http://bmjopen.bmj.com/site/about/guidelines.xhtml

25. Barr B, Clayton S, Whitehead M, Thielen K, Burström B, Nylén L, et al. To what extent have relaxed eligibility requirements and increased generosity of disability benefits acted as disincentives for employment? A systematic review of evidence from countries with well-developed welfare systems. Journal of Epidemiology & Community Health. 2010;64(12):1106-14.
26. Higgins JP, Green S. Cochrane handbook for systematic reviews of interventions: John Wiley & Sons; 2011.
27. Lin L, Chu H. Quantifying publication bias in meta-analysis. Biometrics. 2018;74(3):785-94.
28. Deeks J, Higgins JP, Altman D. Analysing data and undertaking meta-analyses. In: Higgins J, Thomas J, Chandler J, Cumpston M, Li T, Page M, et al., editors. Cochrane Handbook for Systematic Reviews of Interventions version 6. 2019.
29. Popay J, Roberts H, Sowden A, Petticrew M, Arai L, Rodgers M, et al. Guidance on the conduct of narrative synthesis in systematic reviews. A product from the ESRC methods programme. 2006;1:b92.
30. McAllister A, Fritzell S, Almroth M, Harber-Aschan L, Larsson S, Burstrom B. How do macro-level structural determinants affect inequalities in mental health? - a systematic review of the literature. Int J Equity Health. 2018;17(1):180.
31. Bambra C, Gibson M, Sowden A, Wright K, Whitehead M, Petticrew M. Tackling the wider social determinants of health and health inequalities: evidence from systematic reviews. Journal of Epidemiology and Community Health. 2010;64(4):284-91.
32. Naik Y, Baker P, Ismail SA, Tillmann T, Bash K, Quantz D, et al. Going upstream - an umbrella review of the macroeconomic determinants of health and health inequalities. BMC public health. 2019;19(1):1678.

For peer review only

For peer review only - http://bmjopen.bmj.com/site/about/guidelines.xhtml

Reporting checklist for protocol of a systematic review.

Based on the PRISMA-P guidelines.

Instructions to authors

Complete this checklist by entering the page numbers from your manuscript where readers will find each of the items listed below.

Your article may not currently address all the items on the checklist. Please modify your text to include the missing information. If you are certain that an item does not apply, please write "n/a" and provide a short explanation.

Upload your completed checklist as an extra file when you submit to a journal.

In your methods section, say that you used the PRISMA-Preporting guidelines, and cite them as:

Moher D, Shamseer L, Clarke M, Ghersi D, Liberati A, Petticrew M, Shekelle P, Stewart LA. Preferred Reporting Items for Systematic Review and Meta-Analysis Protocols (PRISMA-P) 2015 statement. Syst Rev. 2015;4(1):1.

		Reporting Item	Page Number
**Title**			
Identification	#1a	Identify the report as a protocol of a systematic review	1
Update	#1b	If the protocol is for an update of a previous systematic review, identify as such	1
**Registration**			
	#2	If registered, provide the name of the registry (such as PROSPERO) and registration number	2
**Authors**			
Contact	#3a	Provide name, institutional affiliation, e-mail address of all protocol authors; provide physical mailing address of corresponding author	1

For peer review only

Contribution	#3b	Describe contributions of protocol authors and identify the guarantor of the review	9
**Amendments**			
	#4	If the protocol represents an amendment of a previously completed or published protocol, identify as such and list changes; otherwise, state plan for documenting important protocol amendments	1
**Support**			
Sources	#5a	Indicate sources of financial or other support for the review	9
Sponsor	#5b	Provide name for the review funder and / or sponsor	9
Role of sponsor or funder	#5c	Describe roles of funder(s), sponsor(s), and / or institution(s), if any, in developing the protocol	9
**Introduction**			
Rationale	#6	Describe the rationale for the review in the context of what is already known	3
Objectives	#7	Provide an explicit statement of the question(s) the review will address with reference to participants, interventions, comparators, and outcomes (PICO)	4,5
**Methods**			
Eligibility criteria	#8	Specify the study characteristics (such as PICO, study design, setting, time frame) and report characteristics (such as years considered, language, publication status) to be used as criteria for eligibility for the review	4,5
Information sources	#9	Describe all intended information sources (such as electronic databases, contact with study authors, trial registers or other grey literature sources) with planned dates of coverage	6
Search strategy	#10	Present draft of search strategy to be used for at least one electronic database, including planned limits, such that it could be repeated	9 (Suppl 2)

For peer review only

Study records - data management	#11a	Describe the mechanism(s) that will be used to manage records and data throughout the review	6,7
Study records - selection process	#11b	State the process that will be used for selecting studies (such as two independent reviewers) through each phase of the review (that is, screening, eligibility and inclusion in meta-analysis)	6,7
Study records - data collection process	#11c	Describe planned method of extracting data from reports (such as piloting forms, done independently, in duplicate), any processes for obtaining and confirming data from investigators	6,7
Data items	#12	List and define all variables for which data will be sought (such as PICO items, funding sources), any pre-planned data assumptions and simplifications	7
Outcomes and prioritization	#13	List and define all outcomes for which data will be sought, including prioritization of main and additional outcomes, with rationale	5,6
Risk of bias in individual studies	#14	Describe anticipated methods for assessing risk of bias of individual studies, including whether this will be done at the outcome or study level, or both; state how this information will be used in data synthesis	7
Data synthesis	#15a	Describe criteria under which study data will be quantitatively synthesised	7,8
Data synthesis	#15b	If data are appropriate for quantitative synthesis, describe planned summary measures, methods of handling data and methods of combining data from studies, including any planned exploration of consistency (such as I2, Kendall's τ)	7
Data synthesis	#15c	Describe any proposed additional analyses (such as sensitivity or subgroup analyses, meta-regression)	7
Data synthesis	#15d	If quantitative synthesis is not appropriate, describe the type of summary planned	7
Meta-bias(es)	#16	Specify any planned assessment of meta-bias(es) (such as publication bias across studies, selective reporting within studies)	7

For peer review only

Confidence in cumulative evidence	#17	Describe how the strength of the body of evidence will be assessed (such as GRADE)	7

The PRISMA-P checklist is distributed under the terms of the Creative Commons Attribution License CC-BY 4.0. This checklist was completed on 25. November 2019 using https://www.goodreports.org/, a tool made by the EQUATOR Network in collaboration with Penelope.ai

For peer review only

Supplementary File 2

Preliminary search strategy

Medline

1 austerity.mp.

2 welfare benefit.mp.

3 welfare reform.mp.

4 Social assistance.mp.

5 exp Social Security/

6 Social security.mp.

7 Income benefits.mp.

8 Income support.mp.

9 Income supplement.mp.

10 Income maintenance.mp.

11 Pensions/

12 Conditional cash.mp.

13 Cash assistance.mp.

14 Unemployment benefit.mp.

15 Child Benefit.mp.

16 Tax credit.mp.

17 Family benefit.mp.

18 Family support.mp.

19 Conditionality.mp.

20 Poverty reduction.mp.

21 Housing benefit.mp.

22 Anti-poverty.mp.

23 Family allowance.mp.

24 Entitlement.mp.

25 Generosity.mp.

26 Disability benefit.mp.

27 1 or 2 or 3 or 4 or 5 or 6 or 7 or 8 or 9 or 10 or 11 or 12 or 13 or 14 or 15 or 16 or 17 or 18 or 19 or 20 or 21 or 22 or 23 or 24 or 25 or 26

For peer review only

28 Mental.mp.

29 Psychiat*.mp.

30 Distress*.mp.

31 Stress*.mp.

32 Depress*.mp.

33 exp Depression/

34 Wellbeing.mp.

35 Well-being.mp.

36 Anxiety.mp.

37 Quality of life.mp.

38 Mood disorder.mp.

39 Psychological.mp.

40 Suici*.mp.

41 Self-harm.mp.

42 28 or 29 or 30 or 31 or 32 or 33 or 34 or 35 or 36 or 37 or 38 or 39 or 40 or 41

43 Quantitative.mp.

44 Natural experiment.mp.

45 Quasi-experimental.mp.

46 Difference-in-difference.mp.

47 Multi-level.mp.

48 Regression.mp.

49 Longitudinal.mp.

50 Time-series.mp.

51 Logistic.mp.

52 Linear.mp.

53 Poisson.mp.

54 Synthetic control.mp.

55 propensity score.mp.

56 Instrumental variable.mp.

57 Decomposition.mp.

For peer review only

58 Cross-sectional.mp.

59 (before adj2 after).mp.

60 (pre adj2 post).mp.

61 Quasi-natural.mp.

62 Natural Policy Experiment.mp.

63 Confounding.mp.

64 Fixed-effects.mp.

65 43 or 44 or 45 or 46 or 47 or 48 or 49 or 50 or 51 or 52 or 53 or 54 or 55 or 56 or 57 or 58 or 59 or 60 or 61 or 62 or 63 or 64

66 27 and 42 and 65

For peer review only

Investigating the relationship between changes in social security benefits and mental health: a protocol for a systematic review

Journal:	*BMJ Open*
Manuscript ID	bmjopen-2019-035993.R2
Article Type:	Protocol
Date Submitted by the Author:	06-May-2020
Complete List of Authors:	Simpson, Julija; Newcastle University, Population Health Sciences Institute<br>Brown, Heather ; Newcastle University, Population Health Sciences Institute<br>Bell, Zoe; Newcastle University, Population Health Sciences Institute<br>Albani, Viviana; Newcastle University, Population Health Sciences Institute<br>Bambra, Clare; Newcastle University, Population Health Sciences Institute
**Primary Subject Heading**:	Public health
Secondary Subject Heading:	Mental health
Keywords:	PUBLIC HEALTH, SOCIAL MEDICINE, MENTAL HEALTH

SCHOLARONE™
Manuscripts

I, the Submitting Author has the right to grant and does grant on behalf of all authors of the Work (as defined in the below author licence), an exclusive licence and/or a non-exclusive licence for contributions from authors who are: i) UK Crown employees; ii) where BMJ has agreed a CC-BY licence shall apply, and/or iii) in accordance with the terms applicable for US Federal Government officers or employees acting as part of their official duties; on a worldwide, perpetual, irrevocable, royalty-free basis to BMJ Publishing Group Ltd ("BMJ") its licensees and where the relevant Journal is co-owned by BMJ to the co-owners of the Journal, to publish the Work in this journal and any other BMJ products and to exploit all rights, as set out in our licence.

The Submitting Author accepts and understands that any supply made under these terms is made by BMJ to the Submitting Author unless you are acting as an employee on behalf of your employer or a postgraduate student of an affiliated institution which is paying any applicable article publishing charge ("APC") for Open Access articles. Where the Submitting Author wishes to make the Work available on an Open Access basis (and intends to pay the relevant APC), the terms of reuse of such Open Access shall be governed by a Creative Commons licence – details of these licences and which Creative Commons licence will apply to this Work are set out in our licence referred to above.

Other than as permitted in any relevant BMJ Author's Self Archiving Policies, I confirm this Work has not been accepted for publication elsewhere, is not being considered for publication elsewhere and does not duplicate material already published. I confirm all authors consent to publication of this Work and authorise the granting of this licence.

For peer review only

Investigating the relationship between changes in social security benefits and mental health: a protocol for a systematic review

Julija Simpson*[1], Heather Brown[1], Zoe Bell[1], Viviana Albani[1] and Clare Bambra[1]

*Corresponding author: Julija Simpson: julija.stoniute@newcastle.ac.uk

1 – Population Health Sciences Institute, Newcastle University, Newcastle upon Tyne, NE1 4LP

Word Count: 3,283 (excluding references)

Protocol amendments

In the event of protocol amendments, the date of each amendment will be accompanied by a description of the change and the rationale

For peer review only - http://bmjopen.bmj.com/site/about/guidelines.xhtml

Abstract

Introduction. Poor mental health is one of the greatest causes of disability in the world. Evidence increasingly shows that population mental health may be influenced by national social security policies. This systematic review aims to establish the relationship between social security and mental health in order to help inform recommendations for policy makers, practitioners and future research.

Methods and analysis. A systematic review of quantitative observational studies investigating mental health outcomes related to changes in social security policies will be conducted. Six major databases, including Medline, PsychInfo, Embase, Cumulative Index to Nursing and Allied Health Literature (CINAHL), Applied Social Sciences Index Abstracts (ASSIA), Scopus, as well as Research Papers in Economics will be searched from January 1979 to April 2020. The electronic database searches will be supplemented by reference and citation searches as well as hand-searching of key journals. The outcomes of interest are objective or subjective mental health outcomes, including stress, anxiety, depression, self-reported mental health scores, subjective well-being and suicide. Study selection will follow the Preferred Reporting Items for Systematic Reviews and Meta-Analyses guidelines, and the quality of the studies will be assessed by the Validity Assessment Framework designed for appraising econometric studies. A narrative synthesis will be conducted for all included studies. If data permit, study findings will be synthesised by conducting a meta-analysis.

Ethics and dissemination. As it will be a systematic review, without primary data collection, there will be no requirement for ethical approval. Findings will be disseminated through peer-reviewed publication and in various media, for example, conferences or symposia.

Trial registration number International Prospective Register for Systematic Reviews (PROSPERO) number CRD42019154733.

Strengths and limitations of this study

- We employ a rigorous international gold standard methodology of reporting (PRISMA-P) to facilitate the development and reporting of this protocol
- By employing a PRISMA-P guideline this study follows a clear framework to improve the transparency, accuracy and completeness of the systematic review protocol
- To ensure comprehensiveness of the evidence base in the review, we have developed a broad search strategy, supplemented with reference and citation searches as well as hand-searching of key journals
- A potential limitation of this study is that studies may be too heterogeneous to combine effect estimates

Keywords

Mental health, social security, welfare reform, systematic review

For peer review only

Introduction

Mental illness is a major cause of mortality and morbidity worldwide (1). In OECD (Organisation for Economic Cooperation and Development) countries, mental ill-health affects an estimated 20 per cent of the working-age population at any given time, causing major losses to individuals, communities and the economy (2). It has been estimated that the global economy loses about $1 trillion every year in productivity as a result of depression and anxiety (3).

Evidence increasingly shows that mental ill-health is socially determined. (4, 5). The social determinants include "the conditions in which people are born, live, work and age, and the health systems they can access, which are, in turn, shaped by a wider set of forces: economics, social, environmental policies, and politics" (6, p.392).

One of the key policies affecting household income and thus living conditions – particularly those in the lowest socio-economic groups - is social security policy. Broadly defined, social security policy includes income transfers from the state to individuals, with the aim to cover income loss due to poor health and disability, unemployment, low income, single parenthood and old age (7).

A growing body of literature indicates that, while not explicitly designed to, social security policies can have an impact on population mental health. For example, suicide rates in men in the USA fall sharply when individuals become eligible for social security retirement benefits, even though only a small fraction of men retire at this age (8). Moreover, two major systematic reviews investigating the relationship between income and health in OECD countries also found that additional financial resources, including those from social security benefits, can have positive effects on both adult (9) and child mental health (10, 11). Lack of social protection, on the other hand, has been associated with negative effects on mental health. In the UK, the austerity-driven changes to the social security system have been associated with increased prevalence of depression (12), suicide (13) and mental health disorders (14).

Although there is a growing body of literature documenting strong links between social disadvantage and mental health problems (15, 16), the evidence base on the health impact of social security reform remains small. A recent umbrella review of social protection policies in OECD countries has identified only two low-quality systematic reviews related to income maintenance policies (17). Both of these reviews focused on specific policies (unemployment benefits and tax credits) and, to date, the broader range of social security policies and their impacts on mental health has not been reviewed. Furthermore, even though there have been broad systematic reviews of conditional cash transfers in low- and middle-income countries (18, 19), to our knowledge, none of these reviews included mental health as an outcome. Therefore, in the context of global mental health crisis as well as rapidly rising concerns about the impact of recent austerity measures on population health and wellbeing, there is a growing need to better understand the relationship between social security reform and mental health.

The aim of this systematic review is to evaluate the impact of social security reform on mental health and mental health inequalities in high-income countries. By providing robust and concrete evidence, this review will add to the growing evidence base linking social

For peer review only

For peer review only - http://bmjopen.bmj.com/site/about/guidelines.xhtml

determinants and mental health and will help inform recommendations for policy makers, researchers and areas for further research.

Methods

The review protocol is reported in accordance with the standards of the Preferred Reporting Items for Systematic Reviews and Meta-analysis Protocols (20) (PRISMA-P, see supplementary file 1).

Preliminary searchers were carried out in October 2019 and the study registered with the PROSPERO International Prospective Register of Systematic Reviews (21) (registration number CRD42019154733) on 31 October 2019. The review is planned to commence February 2020 and is anticipated to take nine months to complete.

Systematic review questions

1. What is the impact of social security reform on mental health?
2. What is the impact of social security reform on mental health and mental health inequalities?

Mental health inequalities are defined as differences in mental health effects by commonly used indicators of socio-economic status (SES) such as individual income, wealth, poverty, education level, occupational status, welfare benefit receipt; as well as area-level economic indicators and ethnicity given the strong relationship between ethnicity and lower SES particularly in the USA (22) – as defined by Hillier-Brown et al (17).

Objectives

This study has two objectives:

1. To systematically review the quantitative evidence of changes in social security benefit policies on mental health and mental health inequalities;
2. To establish the associational pathways linking social security benefits and mental health.

Eligibility criteria

Studies will be selected according to the criteria outlined in Table 1.

Table 1 Eligibility Criteria

	**Inclusion**	**Exclusion**
Participants (P)	All individuals of any age	
Intervention (I)	A national or regional level social security reform or a series of reforms, defined as any government change to:<br>1. Cash benefit levels<br>2. Eligibility/conditionality related to benefit receipt (including changes in eligibility assessment policies)	Interventions (i.e. not government policies such as employer-funded insurance schemes)<br><br>Multi-sectorial policies (e.g. Active Labour Market Programmes, broad austerity policies, welfare regimes)

For peer review only

	3. Introduction or elimination of a benefits policy	Policies not considering cash transfers (e.g. in-kind benefits)<br><br>Parental leave, child-care subsidy policies
Comparison (C)	With or without a clearly defined control group	
Outcomes (O)	Clearly defined mental health outcomes (e.g. depression, anxiety, stress, suicide), and could include subjective measures, such as subjective well-being and life satisfaction, as well as symptoms, events and diagnoses<br><br>Emotional and behavioural aspects of child wellbeing	Non-mental health outcomes (e.g. general health if there is no separate mental health component)<br><br>Outcomes related to health-care utilisation (e.g. hospital admissions, anti-depressant prescriptions)
Study type	Observational studies evaluating change(s) to a specific policy, including:<br><br>Cohort studies<br><br>Cross-sectional studies<br><br>Longitudinal studies<br><br>Quasi-experimental studies	Descriptive studies reporting mental health outcomes in benefit recipients vs non recipients or between different recipient groups<br><br>Randomised controlled trials<br><br>Qualitative studies<br><br>Editorials<br><br>Commentaries<br><br>Expert opinion articles
Study period	Published in the last 40 years (1979-2020)	Literature published before 1979
Setting	High-income countries (as per World Bank definition*)	Non-high income countries
Study reporting language	English	

Interventions

The review will examine a range of social security policies that cover income loss due to poor health and disability, unemployment, low income, single parenthood and old age as well as tax credits and benefits for families with dependent children.

For peer review only - http://bmjopen.bmj.com/site/about/guidelines.xhtml

Outcomes

Primary

Studies will be included if one of the outcome measures reported is the number or rate of mental health problems reported. Mental health can be measured both objectively and subjectively. Objective measures include formal diagnoses (e.g. anxiety, depression, suicide). Subjective measures include those that are self-reported (e.g. self-assessed mental health and wellbeing scores, days of good/poor mental health or by validated questionnaires such as Warwick-Edinburgh Mental Health Scale. For children, outcomes relating to emotional and behavioural aspects of well-being will be included.

Outcomes will be collected as reported. Due to possible variation in disease definitions over time, we will extract definitions of outcomes as reported in individual studies. We will extract outcomes in all data forms (e.g. dichotomous, continuous) as reported in the included studies.

Studies including general self-assessed health will not be included unless they also report a mental health component. Outcomes reported for specific population subgroups (e.g. by socio-economic status) will also be included. There will be no restrictions on timing of outcomes.

Secondary

Secondary outcomes of this study include mental health inequalities, as measured by socio-economic status.

Study exclusion

Studies will be excluded they were published prior to 1979. The year 1979 was selected as a cut-off date because it marks a significant shift in policy from this period onwards going from passive to active welfare in high-income countries. Only studies in high income countries will be included.

Study design

Observational study designs will be included if they report mental health outcomes related to a national (or regional) social security policy change and are based on a quantitative method.

Search Strategy

We will examine seven major databases, including MEDLINE, EMBASE, CINAHL, ASSIA, PsycINFO and Research Papers in Economics from January 1979 until April 2020.

Search terms were identified from scoping searches and comprise three main concepts related to mental health, social security policy change and a quantitative research method. While not exhaustive, the search strategy aims to cover a broad range of social security policies, mental health outcomes and quantitative research methods. An information specialist, trained in systematic reviews, as well as a medical librarian were consulted about designing and piloting the search strategy. A sample search strategy adapted for Medline can be found in Supplementary File 2.

In terms of restrictions, only peer-reviewed English language studies will be included. One exception includes working-papers in the field of Economics, as they may comprise a large proportion of relevant literature. Electronic database searches will be supplemented by evaluating the references of literature included in the review as well as the references of other major reviews in the field. Citation searches of included articles will be undertaken using the

For peer review only - http://bmjopen.bmj.com/site/about/guidelines.xhtml

Science and Social Science Citation Indices. Hand-searching of key journals on health and social policy will also be conducted.

Study Selection

Studies will be imported to EndNote (version X9) (23) for deduplication and then to an online software programme, Rayyan (24) for screening. One reviewer (JS) will screen all the titles and abstracts, and, given the large numbers of the initial search hits, the second reviewer (ZB) will screen a random sample of 10% of these, with a third reviewer assisting to resolve any disagreements. We will obtain full reports for all titles that appear to meet the inclusion criteria or where there is any uncertainty. If necessary, additional information will be sought by contacting the study authors. Both reviewers will assess the eligibility of the full texts and appraise the quality of the included studies.

Prior to data extraction, a data extraction form will be created and pilot-tested using a sub-set of included studies. The form will be modified based on feedback from data extractors in order to improve the usability and appropriateness of the form. The data abstraction framework will be used as a template for recording significant study characteristics of the literature appropriate for inclusion in the review. This information will include details on: Study author, Year, Title, Population, Mental health outcome measure(s), Policy, Study design, Data source, Data years, Analytic approach and Results. Data will be extracted by two reviewers independently (JS and HB/VA).

Risk of bias assessment

Quality appraisal will be undertaken by two researchers working independently (JS and HB/VA), using the Validity Assessment framework developed by Barr and colleagues (25). It includes nine component rating sections (unit of analysis; comparison approach; sample selection; number of time points of data; response bias; exogeneity of policy exposure, confounding, sample size/power and statistical methods). Based on the performance in terms of the above criteria, each study will be assigned a global score and classed as either 'strong' or 'moderate' or 'weak'. Any disagreements between the researchers will be resolved through a discussion between reviewers, if necessary with a third reviewer (ZB). The quality of the studies will form a part of the narrative synthesis to highlight the variation between studies, and to help demonstrate the overall strength of evidence in this study area.

Data synthesis

Since combining results from individual studies has the advantage of increasing both statistical power and precision in estimating the impact of social security policies, it is beneficial to undertake a meta-analysis of collated studies in circumstances where clinical and methodological homogeneity permit. In the event that sufficient studies of similar construct and outcomes are identified, a meta-analysis of the results will be undertaken. The continuous outcomes relating to mental health will likely be studied by investigation of the policy effect size, through measurement of the standardised mean difference in mental health before and after policy in question. This will be presented in a forest plot, facilitating both visual and statistical comparison. A sensitivity analysis will be undertaken, addressing whether the exclusion of studies with poor quality rating has an impact on the precision and overall conclusions of the review. This will be done by excluding one paper at a time and exploring the impact on the overall results. Heterogeneity will be quantified by measuring the I^2 statistic (I^2 statistic with values of 30 to 60%, 50 to 90% and 75 to 100% used to denote

For peer review only - http://bmjopen.bmj.com/site/about/guidelines.xhtml

moderate, substantial and considerable levels of heterogeneity, respectively (26)). If appropriate, heterogeneity will be further explored by undertaking sub-group analysis. Planned subgroup analyses include those by age, gender and type of policy reform. Publication bias will be assessed using funnel plots, as well as Egger's regression test (27).

If meta-analysis is not possible, mental health effects of each policy will be summarised narratively as follows. First, policies will be classified into five broad categories of social security benefits (i.e. Social Assistance; Families with Children; Disability; Unemployment; and Retirement). Policies will be further sub-divided by type of reform – as changes to either benefit levels, eligibility or introduction/elimination of policy. Different magnitudes of each policy will be taken into account by assigning them differential weights, using a modified version of 'direct weighting' approach recommended the Cochrane group (28). This will entail assigning each policy different weights depending on: affected population size; change in magnitude in terms of either benefit level, eligibility requirements or the scale of the policy introduced/removed; risk of bias in each study. The direction of effects will be broadly summarised as either more restrictive or generous policy.

In addition, we will present a tabulated summary of key study characteristics which will include a description of policies, outcomes, populations and settings. This will be followed by a clear accompanying descriptive account addressing the robustness of the evidence presented, and the relationships within and between studies included in the review. Since by definition, narrative synthesis is an inherently more subjective process, it is crucial to utilise a rigorous and transparent approach to the data collected. For this reason, we will use The Economic and Social Research Council framework and guidelines to present the narrative synthesis, as described by Popay and colleagues (29). More specifically, this will include developing a logic model presenting the potential pathways and mediating factors between changes in social security benefits and mental health (e.g. income and employment). As such, the logic model will help guide the structure and interpretation of the findings of the review.

Patient and public involvement

No patients or public are directly involved in this study. The data for systematic review will be collected from previously published studies.

Discussion

Strengths and limitations

To our knowledge, this will be the first systematic review to explore the relationship between social security reform and mental health. Building on a number of successful reviews linking social determinants and health (17, 30-32), the wide range of policies included will provide a comprehensive overview of how social security policies can affect mental health. In our view, taking a comprehensive overview is important because social security or 'welfare' reform usually constitutes a number of policies and thus cannot be reduced to a single intervention. The review will therefore provide valuable information for policymakers as well as insights for areas for future research. In particular, it will help identify gaps in terms of what types of policies require further investigation. Lastly, by following a systematic and transparent approach, and adhering to recommended and validated methods guidelines, we aim to ensure that our findings present a valid representation of the existing evidence.

For peer review only - http://bmjopen.bmj.com/site/about/guidelines.xhtml

There are several potential limitations associated with this review. First, including a wide range of policies and mental health outcomes, may result in a high heterogeneity between included studies which may limit the statistical power to conduct a meta-analysis. Second, our decision to exclude non-English studies, might exclude some relevant policy evaluations, particularly those outside of Europe, Australia or North America. However, due to resource limitations, this is beyond the scope of our review. Further, as with most systematic reviews, our review is susceptible to publication bias whereby only studies with non-null findings are more likely to be published. We aim to minimise this limitation by reviewing the references and citations of the included studies as well as by hand-searching the key journals and including economic working papers, in this way ensuring that the broadest range of relevant literature has been identified. The findings of our review may also limited by the nature of study designs of the included studies, as they will be observational. We will however conduct a rigorous quality assessment of each study and will account for study quality in our narrative synthesis.

Ethics and Dissemination

Since this is a review of secondary data, ethical approval will not be needed. The findings from this review will be disseminated by submission to a peer-reviewed journal. The results will also be presented at a national conference and circulated to the general public and key stakeholder groups using social media.

Author Contributions

JS drafted the manuscript and is the guarantor for the review. All authors contributed to the development of the selection criteria, the risk of bias assessment strategy and data extraction criteria. JS developed the search strategy, HB, ZB, VA and CB provided comments and amendments. All authors read, provided feedback and approved the final manuscript.

Funding Statement

This systematic review is funded by the Economic and Social Research Council, as a part of a PhD project. The funder had no role in developing the systematic review protocol.

Conflicts of interest

None declared.

Abbreviations

PRISMA-P: Preferred Reporting Items for Systematic Reviews and Meta-Analyses (for Protocols)

PROSPERO: international prospective register of systematic reviews

Supplementary Files

Supplementary File 1 – completed PRISMA-P checklist

Supplementary File 2 – preliminary search strategy (Medline)

For peer review only - http://bmjopen.bmj.com/site/about/guidelines.xhtml

Bibliography

1. Vos T, Barber RM, Bell B, Bertozzi-Villa A, Biryukov S, Bolliger I, et al. Global, regional, and national incidence, prevalence, and years lived with disability for 301 acute and chronic diseases and injuries in 188 countries, 1990–2013: a systematic analysis for the Global Burden of Disease Study 2013. The Lancet. 2015;386(9995):743-800.
2. World Bank Group. Mental Health 2018. Available from: https://www.worldbank.org/en/topic/mental-health (accessed 11/02/2020).
3. Chisholm D, Sweeny K, Sheehan P, Rasmussen B, Smit F, Cuijpers P, et al. Scaling-up treatment of depression and anxiety: a global return on investment analysis. The Lancet Psychiatry. 2016;3(5):415-24.
4. Allen J, Balfour R, Bell R, Marmot M. Social determinants of mental health. International Review of Psychiatry. 2014;26(4):392-407.
5. Macintyre A, Ferris D, Gonçalves B, Quinn N. What has economics got to do with it? The impact of socioeconomic factors on mental health and the case for collective action. Palgrave Communications. 2018;4(1):10.
6. Marmot M, Friel S, Bell R, Houweling TA, Taylor S, Health Commission on Social Determinants of Health. Closing the gap in a generation: health equity through action on the social determinants of health. The Lancet. 2008;372(9650):1661-9.
7. Bambra C. Work, worklessness, and the political economy of health: Oxford University Press; 2011.
8. DeSimone J. Suicide and the social security early retirement age. Contemporary economic policy. 2018;36(3):435-50.
9. Cooper K, Stewart K. Does money in adulthood affect adult outcomes. 2015.
10. Cooper K, Stewart K. Does money affect children's outcomes? 2013.
11. Cooper K. Kitty Stewart Does Money Affect Children's Outcomes? An update. 2017.
12. Reeves A, Clair A, McKee M, Stuckler D. Reductions in the United Kingdom's Government Housing Benefit and Symptoms of Depression in Low-Income Households. American Journal of Epidemiology. 2016;184(6):421-9.
13. Barr B, Taylor-Robinson D, Stuckler D, Loopstra R, Reeves A, Whitehead M. 'First, do no harm': are disability assessments associated with adverse trends in mental health? A longitudinal ecological study. Journal of Epidemiology and Community Health. 2016;70(4):339-45.
14. Katikireddi SV, Molaodi OR, Gibson M, Dundas R, Craig P. Effects of restrictions to Income Support on health of lone mothers in the UK: a natural experiment study. Lancet Public Health. 2018;3(7):e333-e40.
15. Fryers T, Melzer D, Jenkins R. Social inequalities and the common mental disorders. Social psychiatry and psychiatric epidemiology. 2003;38(5):229-37.
16. Muntaner C, Eaton WW, Miech R, O'Campo P. Socioeconomic position and major mental disorders. Epidemiologic reviews. 2004;26(1):53-62.
17. Hillier-Brown F, Thomson K, McGowan V, Cairns J, Eikemo TA, Gil-Gonzále D, et al. The effects of social protection policies on health inequalities: evidence from systematic reviews. Scandinavian journal of public health. 2019;47(6):655-65.
18. Bastagli F, Hagen-Zanker J, Harman L, Barca V, Sturge G, Schmidt T. The impact of cash transfers: A review of the evidence from low-and middle-income countries. Journal of Social Policy. 2019;48(3):569-94.
19. Lagarde M, Haines A, Palmer N. The impact of conditional cash transfers on health outcomes and use of health services in low and middle income countries. The Cochrane database of systematic reviews. 2009(4):Cd008137.

For peer review only - http://bmjopen.bmj.com/site/about/guidelines.xhtml

20. Shamseer L, Moher D, Clarke M, Ghersi D, Liberati A, Petticrew M, et al. Preferred reporting items for systematic review and meta-analysis protocols (PRISMA-P) 2015: elaboration and explanation. Bmj. 2015;349:g7647.
21. PROSPERO. York, England: Centre for Reviews and Dissemination; University of York; 2019. Available from: https://www.crd.york.ac.uk/prospero/ (accessed 11/02/2020).
22. Williams DR, Collins C. US socioeconomic and racial differences in health: patterns and explanations. Annual review of sociology. 1995;21(1):349-86.
23. Web of Science Group. Endnote X9 2019. Available from: https://endnote.com/(accessed 11/02/2020).
24. Ouzzani M, Hammady H, Fedorowicz Z, Elmagarmid A. Rayyan—a web and mobile app for systematic reviews. Systematic reviews. 2016;5(1):210.
25. Barr B, Clayton S, Whitehead M, Thielen K, Burström B, Nylén L, et al. To what extent have relaxed eligibility requirements and increased generosity of disability benefits acted as disincentives for employment? A systematic review of evidence from countries with well-developed welfare systems. Journal of Epidemiology & Community Health. 2010;64(12):1106-14.
26. Higgins JP, Green S. Cochrane handbook for systematic reviews of interventions: John Wiley & Sons; 2011.
27. Lin L, Chu H. Quantifying publication bias in meta-analysis. Biometrics. 2018;74(3):785-94.
28. Deeks J, Higgins JP, Altman D. Analysing data and undertaking meta-analyses. In: Higgins J, Thomas J, Chandler J, Cumpston M, Li T, Page M, et al., editors. Cochrane Handbook for Systematic Reviews of Interventions version 6. 2019.
29. Popay J, Roberts H, Sowden A, Petticrew M, Arai L, Rodgers M, et al. Guidance on the conduct of narrative synthesis in systematic reviews. A product from the ESRC methods programme. 2006;1:b92.
30. McAllister A, Fritzell S, Almroth M, Harber-Aschan L, Larsson S, Burstrom B. How do macro-level structural determinants affect inequalities in mental health? - a systematic review of the literature. Int J Equity Health. 2018;17(1):180.
31. Bambra C, Gibson M, Sowden A, Wright K, Whitehead M, Petticrew M. Tackling the wider social determinants of health and health inequalities: evidence from systematic reviews. Journal of Epidemiology and Community Health. 2010;64(4):284-91.
32. Naik Y, Baker P, Ismail SA, Tillmann T, Bash K, Quantz D, et al. Going upstream - an umbrella review of the macroeconomic determinants of health and health inequalities. BMC public health. 2019;19(1):1678.

For peer review only

For peer review only - http://bmjopen.bmj.com/site/about/guidelines.xhtml

Reporting checklist for protocol of a systematic review.

Based on the PRISMA-P guidelines.

Instructions to authors

Complete this checklist by entering the page numbers from your manuscript where readers will find each of the items listed below.

Your article may not currently address all the items on the checklist. Please modify your text to include the missing information. If you are certain that an item does not apply, please write "n/a" and provide a short explanation.

Upload your completed checklist as an extra file when you submit to a journal.

In your methods section, say that you used the PRISMA-Preporting guidelines, and cite them as:

Moher D, Shamseer L, Clarke M, Ghersi D, Liberati A, Petticrew M, Shekelle P, Stewart LA. Preferred Reporting Items for Systematic Review and Meta-Analysis Protocols (PRISMA-P) 2015 statement. Syst Rev. 2015;4(1):1.

		Reporting Item	Page Number
**Title**			
Identification	#1a	Identify the report as a protocol of a systematic review	1
Update	#1b	If the protocol is for an update of a previous systematic review, identify as such	1
**Registration**			
	#2	If registered, provide the name of the registry (such as PROSPERO) and registration number	2
**Authors**			
Contact	#3a	Provide name, institutional affiliation, e-mail address of all protocol authors; provide physical mailing address of corresponding author	1

For peer review only

Contribution	#3b	Describe contributions of protocol authors and identify the guarantor of the review	9
**Amendments**			
	#4	If the protocol represents an amendment of a previously completed or published protocol, identify as such and list changes; otherwise, state plan for documenting important protocol amendments	1
**Support**			
Sources	#5a	Indicate sources of financial or other support for the review	9
Sponsor	#5b	Provide name for the review funder and / or sponsor	9
Role of sponsor or funder	#5c	Describe roles of funder(s), sponsor(s), and / or institution(s), if any, in developing the protocol	9
**Introduction**			
Rationale	#6	Describe the rationale for the review in the context of what is already known	3
Objectives	#7	Provide an explicit statement of the question(s) the review will address with reference to participants, interventions, comparators, and outcomes (PICO)	4,5
**Methods**			
Eligibility criteria	#8	Specify the study characteristics (such as PICO, study design, setting, time frame) and report characteristics (such as years considered, language, publication status) to be used as criteria for eligibility for the review	4,5
Information sources	#9	Describe all intended information sources (such as electronic databases, contact with study authors, trial registers or other grey literature sources) with planned dates of coverage	6
Search strategy	#10	Present draft of search strategy to be used for at least one electronic database, including planned limits, such that it could be repeated	9 (Suppl 2)

For peer review only

Study records - data management	#11a	Describe the mechanism(s) that will be used to manage records and data throughout the review	6,7
Study records - selection process	#11b	State the process that will be used for selecting studies (such as two independent reviewers) through each phase of the review (that is, screening, eligibility and inclusion in meta-analysis)	6,7
Study records - data collection process	#11c	Describe planned method of extracting data from reports (such as piloting forms, done independently, in duplicate), any processes for obtaining and confirming data from investigators	6,7
Data items	#12	List and define all variables for which data will be sought (such as PICO items, funding sources), any pre-planned data assumptions and simplifications	7
Outcomes and prioritization	#13	List and define all outcomes for which data will be sought, including prioritization of main and additional outcomes, with rationale	5,6
Risk of bias in individual studies	#14	Describe anticipated methods for assessing risk of bias of individual studies, including whether this will be done at the outcome or study level, or both; state how this information will be used in data synthesis	7
Data synthesis	#15a	Describe criteria under which study data will be quantitatively synthesised	7,8
Data synthesis	#15b	If data are appropriate for quantitative synthesis, describe planned summary measures, methods of handling data and methods of combining data from studies, including any planned exploration of consistency (such as I2, Kendall's τ)	7
Data synthesis	#15c	Describe any proposed additional analyses (such as sensitivity or subgroup analyses, meta-regression)	7
Data synthesis	#15d	If quantitative synthesis is not appropriate, describe the type of summary planned	7
Meta-bias(es)	#16	Specify any planned assessment of meta-bias(es) (such as publication bias across studies, selective reporting within studies)	7

For peer review only

Confidence in cumulative evidence	#17	Describe how the strength of the body of evidence will be assessed (such as GRADE)	7

The PRISMA-P checklist is distributed under the terms of the Creative Commons Attribution License CC-BY 4.0. This checklist was completed on 25. November 2019 using https://www.goodreports.org/, a tool made by the EQUATOR Network in collaboration with Penelope.ai

For peer review only

Supplementary File 2

Preliminary search strategy

Medline

1 austerity.mp.

2 welfare benefit.mp.

3 welfare reform.mp.

4 Social assistance.mp.

5 exp Social Security/

6 Social security.mp.

7 Income benefits.mp.

8 Income support.mp.

9 Income supplement.mp.

10 Income maintenance.mp.

11 Pensions/

12 Conditional cash.mp.

13 Cash assistance.mp.

14 Unemployment benefit.mp.

15 Child Benefit.mp.

16 Tax credit.mp.

17 Family benefit.mp.

18 Family support.mp.

19 Conditionality.mp.

20 Poverty reduction.mp.

21 Housing benefit.mp.

22 Anti-poverty.mp.

23 Family allowance.mp.

24 Entitlement.mp.

25 Generosity.mp.

26 Disability benefit.mp.

27 1 or 2 or 3 or 4 or 5 or 6 or 7 or 8 or 9 or 10 or 11 or 12 or 13 or 14 or 15 or 16 or 17 or 18 or 19 or 20 or 21 or 22 or 23 or 24 or 25 or 26

For peer review only

28 Mental.mp.
29 Psychiat*.mp.
30 Distress*.mp.
31 Stress*.mp.
32 Depress*.mp.
33 exp Depression/
34 Wellbeing.mp.
35 Well-being.mp.
36 Anxiety.mp.
37 Quality of life.mp.
38 Mood disorder.mp.
39 Psychological.mp.
40 Suici*.mp.
41 Self-harm.mp.
42 28 or 29 or 30 or 31 or 32 or 33 or 34 or 35 or 36 or 37 or 38 or 39 or 40 or 41
43 Quantitative.mp.
44 Natural experiment.mp.
45 Quasi-experimental.mp.
46 Difference-in-difference.mp.
47 Multi-level.mp.
48 Regression.mp.
49 Longitudinal.mp.
50 Time-series.mp.
51 Logistic.mp.
52 Linear.mp.
53 Poisson.mp.
54 Synthetic control.mp.
55 propensity score.mp.
56 Instrumental variable.mp.
57 Decomposition.mp.

For peer review only

58 Cross-sectional.mp.

59 (before adj2 after).mp.

60 (pre adj2 post).mp.

61 Quasi-natural.mp.

62 Natural Policy Experiment.mp.

63 Confounding.mp.

64 Fixed-effects.mp.

65 43 or 44 or 45 or 46 or 47 or 48 or 49 or 50 or 51 or 52 or 53 or 54 or 55 or 56 or 57 or 58 or 59 or 60 or 61 or 62 or 63 or 64

66 27 and 42 and 65

For peer review only
